# Deciphering the Role of MicroRNAs in Neuroblastoma

**DOI:** 10.3390/molecules27010099

**Published:** 2021-12-24

**Authors:** Vishnu Priya Veeraraghavan, Selvaraj Jayaraman, Gayathri Rengasamy, Ullas Mony, Dhanraj M Ganapathy, Royapuram Veeraragavan Geetha, Durairaj Sekar

**Affiliations:** 1 Centre of Molecular Medicine and Diagnostics (COMManD), Department of Biochemistry, Saveetha Dental College & Hospitals, Saveetha Institute of Medical & Technical Sciences, Saveetha University, Chennai 600 077, India; drvishnupriyav@gmail.com (V.P.V.); gayathri.jaisai@gmail.com (G.R.); ullasmony@gmail.com (U.M.); 2 Department of Prosthodontics, Saveetha Dental College & Hospitals, Saveetha Institute of Medical & Technical Sciences, Saveetha University, Chennai 600 077, India; dhanrajmganapathy@yahoo.co.in; 3 Department of Microbiology, Saveetha Dental College & Hospitals, Saveetha Institute of Medical & Technical Sciences, Saveetha University, Chennai 600 077, India; geetha@saveetha.com; 4 Cellular and Molecular Research Centre, Saveetha Dental College & Hospitals, Saveetha Institute of Medical & Technical Sciences, Saveetha University, Chennai 600 077, India

**Keywords:** neuroblastoma, microRNAs, miRNA inhibition, therapeutic target, cancer

## Abstract

Neuroblastoma (NB) is a type of peripheral sympathetic nervous system cancer that most commonly affects children. It is caused by the improper differentiation of primitive neural crest cells during embryonic development. Although NB occurs for 8% of paediatric cancers, it accounts for 15% of cancer-related deaths. Despite a considerable increase in cytotoxic chemo- and radiotherapy, patients in advanced stages remain virtually incurable. Therefore, there is a desperate necessity for new treatment strategies to be investigated. Accumulating evidence suggested that microRNAs (miRNAs) are a class of non-coding RNAs with 19–25 nucleotides lengths and play a central role in the development of NB carcinogenesis. Fascinatingly, miRNA inhibitors have an antisense property that can inhibit miRNA function and suppress the activity of mature miRNA. However, many studies have addressed miRNA inhibition in the treatment of NB, but their molecular mechanisms and signalling pathways are yet to be analysed. In this study, we impart the current state of knowledge about the role of miRNA inhibition in the aetiology of NB.

## 1. Introduction

Neuroblastoma (NB) is a rare type of solid tumour that arises in the adrenal glands. It is a cancerous tumour made up of undifferentiated neuroectodermal cells that originate from the neural crest. NB is indistinguishable from developing neuroblastic cells in the embryo, as it is typical of an embryonic tumour [1]. NB is one of the most frequent childhood cancers, accounting for around 10% of all cancer-related fatalities in children [2]. Every year approximately 800 new cases of NB are discovered. It affects mostly new-borns and young children, but it can also affect children older than 10 years, and it is more frequent in boys. The initial tumour in NB is frequently found in the abdomen, and risk categories are determined by a variety of clinical and molecular variables [3]. Despite recent therapeutic improvements, continuing clinical trials and basic scientific research, NB remains a difficult medical challenge with an uncertain clinical course and a poor overall prognosis for advanced-stage disease [4]. The degree of cellular differentiation within the tumour determines how NB is classified. NB is a poorly differentiated tumour with an abundance of neuroblasts [5]. NB is currently treated with a combination of surgery, chemotherapy and radiotherapy. Despite recent breakthroughs in treatment choices, the clinical prognosis for aggressive NB remains poor [6]. Many attempts have been made to modify the cellular environment in order to produce safer, more effective therapy choices for NB. With the discovery of microRNAs (miRNAs), a new layer of gene regulation has emerged, providing insights into the complicated pathophysiology of NB that may ultimately help answer outstanding problems.

In general, miRNAs or small non-coding RNAs are single-stranded RNA with a length of 19–25 nucleotides [7]. miRNAs inhibit gene expression by binding to a complementary sequence in the 3’untranslated region of mRNAs, causing them to be targeted for degradation and prevented from being translated [8]. miRNA dysregulation has been documented in a variety of malignancies, with individual miRNAs acting as either tumour promoters or tumour suppressors [9,10]. Profiling research has found that miRNAs are linked to clinical outcomes in NB [11], and specific miRNAs have been discovered to influence essential processes in NB such as apoptosis, differentiation and cell proliferation [12,13]. On the other hand, miRNA inhibitors have the ability to block the target mRNA molecules to suppress the disease progression [14]. The subsequent sections of this paper will go through some of the recent discoveries that have aided in the knowledge of neuroblastoma development and may be used as a therapeutic target.

## 2. Biogenesis of MiRNAs

MiRNAs are short non-coding RNAs with 19–25 nucleotides in length which negatively regulates gene expression at the messenger RNA (mRNA) levels in mammals, plants and some viruses [15]. They have a wide range of expression patterns and have an impact on cellular activities and developmental pathways [16]. The biogenesis of miRNA is a two-step process that includes nuclear and cytoplasmic cleavage. miRNAs are transcribed in the nucleus as a long transcript called primary-miRNAs (pri-miRNA), either by their own promoters or by promoters shared with their host gene [17]. Drosha RNase III endonuclease is responsible for the nuclear cleavage of pri-miRNA. Drosha RNase III endonuclease cleaves both strands of the stem, producing a 60–70 nucleotides stem loop intermediate known as precursor miRNAs (pre-miRNA), which are then exported to the cytoplasm via exportin-5 and Ran-GTP [18]. Pre-miRNAs are processed further in the cytoplasm by another RNase type III enzyme, DICER to produce miRNA duplex [19]. The miRNA duplex is subsequently loaded into an argonaute (AGO) protein, which results in the formation of the RNA-induced silencing complex (RISC), a ribonucleoprotein complex, where one strand is chosen to develop into a mature miRNA [20].

**Figure 1:**
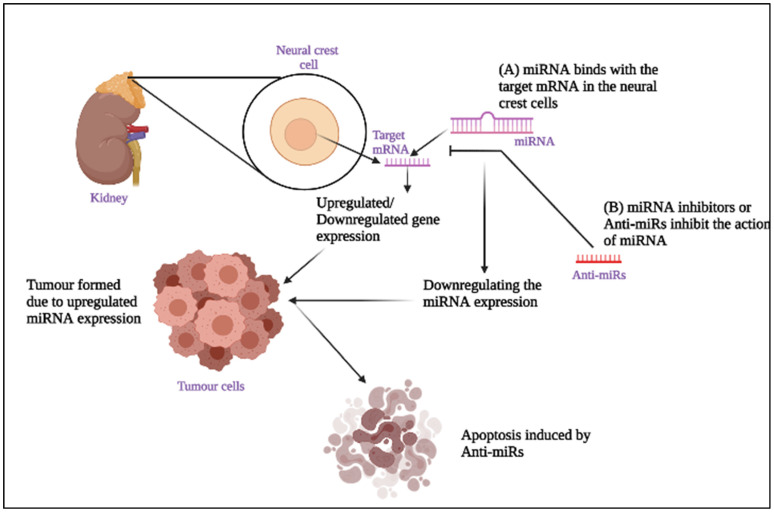
Role of miRNAs and miRNA inhibitor in Neuroblastoma: (**A**) MicroRNAs (miRNAs) with the RISC complex bind to the target mRNAs in the neural crest cells, thus leading to down-regulation or up-regulation of the target gene expression, which leads to a tumour. (**B**) Anti-miRs prevent the action of miRNAs by inhibiting the miRNAs from binding with target mRNA so that down-regulating the miRNA expression leads to apoptosis of the tumour cells.

In addition, the non-canonical miRNA biogenesis pathway also results in the production of functional miRNAs. They are physically and functionally similar to canonical miRNAs, but they have been discovered to follow different maturation pathways, often skipping one or more steps of canonical pathway biogenesis [21]. The mirtron route was the first non-canonical pathway to be found. Deep sequencing data of small RNAs from *Drosophila melanogaster* and *Caenorhabditis elegans* revealed pre-miRNA-sized short introns, which led to the discovery of mirtrons [22,23]. Splicesomes and debranching enzymes in the nucleus, process these introns to form miRNA hairpins that are ready for Dicer cleavage. Exportin-5 then transports the hairpin to the cytoplasm, where Dicer cleaves it. Thus, the mirtron pathway avoids or substitutes microprocessor processing with splicing activity before merging with the standard miRNA pathway at the Exportin-5-bound transport phase [22]. This review mainly focuses on the miRNA inhibition in NB and its role in treatment strategies. Figure 1 represents the role of miRNAs and miRNA inhibitors in Neuroblastoma.

## 3. Molecular Mechanism Involved in NB

Several pathways are involved in the disease progression and inhibition of NB. Amplification of the N-myc gene or mutations of the p53 tumour suppressor genes is common in NB. According to studies, p53 mutations are occasionally linked to neuroblastoma growth, and tumourigenetic consequences of mutant p53 may differ from those of N-myc [24]. In addition, NB can also be caused by MTDH (metadherin) overexpression where MTDH is a transforming downstream mediator of oncogenic Ha-Ras and c-Myc’s activities. Furthermore, MTDH overexpression stimulates proliferation, invasion, cell survival and chemoresistance via activating the PI3K/Akt, nuclear factor kappaB (NFkappaB) and Wnt/beta-catenin signalling pathways [25]. MTDH also promotes metastasis by mediating tumour cell attachment to tissues. Wnt signalling is linked to tumour cell stemness and the production of stem cell markers and is a key component of chemoresistance in NB [26]. Likewise, during embryogenesis, the Ap-2 family has been identified to regulate face, limb and kidney development while also controlling differentiation and apoptosis. Endocrine processes are regulated by these proteins as well. These, when targeted, can lead to tumour progression.

In NB, the NF-κB signalling pathway has an antiapoptotic effect, while IκB kinase (IKK) is a kinase that is required for NF-κB activation. NF-κB is stimulated by IKK phosphorylation. In normal and malignant cells, NF-κB supports cell survival by inducing target genes whose products block components of the apoptotic machinery. By activating genes encoding antioxidant proteins, NF-κB can help prevent programmed necrosis. Many cancer cells, whether epithelial or hematopoietic in origin, use NF-κB to achieve resistance to anticancer drugs, radiation and death cytokines, regardless of mechanism [27]. Moreover, when receptor tyrosine kinases activate PI3K (phosphatidylinositol-3 kinase), it phosphorylates PIP2 (Phosphatidylinositol 4,5-bisphosphate) to create PIP3 (phosphatidylinositol (3,4,5)-trisphosphate) and activates the signalling pathway in NB. PTEN dephosphorylates PIP3 and converts it to PIP2, hence inhibiting the pathway. AKT is a protein that is activated by PIP3 and regulates physiological activities [28]. Meanwhile, ATG5 is a protein that is involved in the early stages of autophagosome production, plays an important role in autophagosome maturation and is linked to chemoresistance in NB [29].

## 4. MiRNAs Down-Regulation in NB

Certain miRNAs that are down-regulated and play an important role in NB are discussed in a study by Ye et al. (2019), which explored the expression and the role of miR-3934-5p in neuroblastoma cell lines and tissue samples. The results revealed that miR-3934-5p was shown to be highly elevated in neuroblastoma tissues and cell lines and *TP53INP1* as a direct target gene of miR-3934-5p. Thus, the overall results confirmed that down-regulation of miR-3934-5p may cause apoptosis and reduce neuroblastoma cell viability. Thus, the recent findings stipulated that miR-3934-5p/*TP53INP1* axis appears to be a unique therapeutic target for neuroblastoma treatment [30]. Engrossingly, the results of Cheng et al. (2019) revealed that in NB cells, miR-34a inhibited cell proliferation, migration, invasion and autophagy while promoting death and miR-34a was found to be a direct target of *ATG5* (autophagy-related gene 5). Furthermore, *ATG5* restoration reduced the inhibitory effect of miR-34a on proliferation, apoptosis, migration, invasion and autophagy. Thus, these results indicated that miR-34a inhibited the progression of NB by targeting *ATG5* and can be used as a novel therapeutic target in treating NB [31]. Wang et al. (2018), suggested that miR-129 and *MYO10* axis-controlled neuroblastoma development and chemosensitivity. *MYO10* expression was decreased by miR-129, which inhibited cell proliferation. miR129-mediated proliferation repression and chemosensitivity were dramatically improved when *MYO10* was re-expressed. Thus, it was concluded that miR-129 suppressed NB growth and increased chemosensitivity by inhibiting *MYO10*, suggesting that it could be a promising target and logical therapy approach for NB [32]. Wu et al. (2015) suggested that miR-362-5p inhibited cell proliferation, migration and invasion of *SH-SY5Y* in both in vivo and in vitro conditions. This study discovered a functional relationship between miR-362-5p and *PI3K-C2b* expression, as well as the fact that *PI3K-C2b* was a direct target of miR-362-5p in NB. Hence, miR-362-5p could be a promising therapeutic option in the treatment of NB [33]. According to the evidence, miR-205 appears to play a role in tumour initiation, growth and metastasis in a variety of malignancies. In human NB tissue samples and cell lines, miR-205 expression was drastically reduced. In contrast, restoring miR-205 in NB cells inhibited cell proliferation, migration and invasion, as well as inducing cell death in vitro and reducing tumour growth in vivo. The direct target gene of miR-205 has been identified as cAMP-responsive element-binding protein 1 (*CREB1*). In NB cells, the expression of *CREB1* and the *CREB1* targets *BCL-2* and *MMP9* (Matrix metalloproteinase9) were drastically reduced when miR-205 was inhibited. The up-regulation of *CREB1* partially reversed the inhibitory effects of miR-205 on NB cells. These findings imply that miR-205 may act as a tumour suppressor in NB via inhibiting *CREB1* [34]. However, cell proliferation is inhibited by miR-338-3p, which also inhibits cell migration and invasion by causing cell cycle arrest by directly targeting *PREX2a*. The PTEN/Akt pathway suppressed cell proliferation, migration and invasion when *PREX2a* was knocked down. This research showed that miR-338-3p inhibits the PTEN/Akt pathway via down-regulating *PREX2a*. Therefore, this newly discovered function of miR-338-3p sheds fresh light on neuroblastoma and may lead to new therapeutic possibilities [35]. A study by Maugeri et al. (2016) on an in vivo murine neuroblastoma progression model revealed that miR-29a-3p and miR-34b-3p expression levels were found to be down-regulated [36]. In addition, it was found that *CDK6*, *DNMT3A* and *DNM3B* are the targets of miR-29a-3p, whereas *CCNE2* and *E2F3* are targets of miR-34b-3p. Thus, the study proposed that miR-29a-3p and miR-34b-3p act as potential therapeutic targets in NB [36]. Table 1 represents the miRNAs and their function in neuroblastoma.

## 5. MiRNAs Up-Regulation in NB

Certain miRNAs that are up-regulated in NB disease and progression are discussed in this section. Overexpression of miR-380-5p, in combination with activated HRAS (Harvey Rat Sarcoma virus) oncoprotein, has been shown to transform primary cells, block oncogene-induced senescence and form tumours in mice, whereas inhibition of endogenous miR380-5p in neuroblastoma cells resulted in p53 induction and extensive apoptotic cell death [38]. Furthermore, overexpression of miR-15a, miR-15b, or miR-16 resulted in a considerable reduction in *MYCN* mRNA and N-Myc protein levels. In NB cells, however, inhibiting miR significantly increased MYCN mRNA and N-Myc protein levels, as well as mRNA half-life. In addition, enhanced expression of miR-15a, miR-15b and miR-16 inhibited NB cell proliferation, migration and invasion. These findings demonstrate that miR-15a, miR-15b and miR-16 target *MYCN* to decrease tumour growth in NB. As a result, these miRs (miR-15a, miR-15b, or miR-16) could be evaluated as prospective NB therapy targets [38]. MiR-145 is well-known for its role as a tumour suppressor in a variety of cancers. Hence, Zhao et al.’s (2020) goal was to look at miR-145 as a potential tumour suppressive effect and mechanisms in high-risk neuroblastoma. In SH-SY-5Y cells, overexpression of miR-145 lowered cell viability and enhanced apoptosis. In SH-SY-5Y cells, it was discovered that *MTDH* (Metadherin) was a direct target for miR-145. Thus, their results revealed that low miR-145 expression is linked to a poor prognosis in NB patients and that overexpression of miR-145 inhibits NB cell proliferation by down-regulating *MTDH*, suggesting that miR-145 could be a target for the development of a microRNA-based NB therapy [39]. A study by Gao et al. (2014) aimed to investigate the significant role and mechanism of miR-200a in neuroblastoma. Overexpression of miR-200a in neuroblastoma cells lowered cell viability and suppressed tumour growth in mice xenografts. It was discovered that AP-2γ to be a novel target for miR-200a. As a result, miR-200a decreases AP-2γ mRNA and protein expression by targeting its 3’UTR. This research discovered that miR-200a could be used as a therapeutic target in treating neuroblastoma by direct targeting AP-2γ [40]. A study by Wu et al. (2018) explored the role and biological process of miR-1247 in NB in vitro conditions. *ZNF346* (Zinc Finger Protein 346) was found to be a target of miR-1247, and its expression could be down-regulated by miR-1247 overexpression. Thus, the study confirmed that miR-1247 is directly targeted to reduce *ZNF346* expression and, hence, decrease the progression of NB, suggesting that it could be a new therapeutic target for NB [41]. Mao et al. (2019) suggested that in NB cells, the overexpression of miR-149 decreased cell proliferation and colony formation while promoting cell death and Dox (doxorubicin) chemosensitivity. MiR-149 also targets cell division cycle 42 (CDC42) and B-cell lymphoma 2 (BCL2). CDC42 and BCL2 mRNA levels were also raised in NB tissues and cells, and restoring CDC42 or BCL2 reduced miR-149’s regulatory influence on NB progression. Thus, the study concluded that miR-149 inhibited cell proliferation and increased Dox chemosensitivity in NB through modulating CDC42 and BCL2, opening up a new therapy option for NB [42].

Zhou et al. (2020) used a nude mouse xenograft model to assess the effect of tumour growth with miR-429 overexpression. IKK mRNA on binding with miR-429 inhibits its role, whereas miR-429 overexpression was found to reduce neuroblastoma growth in a nude mice xenograft model. Therefore, these findings suggested that miR-429 could be a potential target for neuroblastoma treatment by inhibiting cell proliferation, migration and invasion via the NF-κB pathway [43]. Li et al. (2019) confirmed that miR-34a and hepatocyte nuclear factor 4α (HNF4α) expressions in juvenile neuroblastoma tissues. In SH-SY5Y cells, overexpression of miR-34a or knockdown of HNF4α may result in decreased cell proliferation, migration, invasion and MMP-2 and MMP-14 production. It was found that overexpression of HNF4α might reverse the inhibitory effect of miR-34a on SH-SY5Y cell proliferation, migration and invasion. Thus, this study concluded that overexpression of miR-34a, which targets HNF4α, can inhibit SH-SY5Y cells from proliferating, migrating and invading [45]. In NB tissues, it was discovered that miR-203 overexpression decreased the proliferation, migration and invasion of these two cell lines (SK-N-SH and SH-SY5Y NB cells) via binding to the 3’ untranslated region of Sam68. Therefore, the results of this study reported that miR-203/Sam68 could be used as a potential new diagnostic or therapeutic target for NB treatment [46]. Furthermore, Zhang et al. (2012) demonstrated that in both in vivo and in vitro conditions, the overexpression of miR-9 inhibited SH-SY5Y and SK-N-SH cell invasion, metastasis and angiogenesis. NB cells migration, invasion and angiogenesis were prevented by anti-miR-9 or by *MMP-14* knockdown. These findings suggest that miR-9 inhibits neuroblastoma invasion, metastasis and angiogenesis by suppressing MMP-14 production via the 3’-UTR binding site [47]. These findings suggested that miRNA inhibition could be considered as a target for NB treatment. However, our knowledge of the role of miRNA inhibition in NB is still very deficient; thus, due to the limited number and scope, more research is required to know the molecular and signalling pathways of NB.

## 6. Future Perspective and Clinical Relevance

In children, NB is the most frequent extracranial solid cancer. Despite the fact that the molecular basis of neuroblastoma has received a lot of attention in the last decade, unravelling the mechanisms that cause neuroblastoma to develop aggressively is still needed to improve therapy efficacy. miRNAs are short non-coding RNA molecules that target sequence complementarity on the 3′-untranslated regions (3′-UTRs) of certain mRNAs to regulate 60 percent of human gene expression at the post-transcriptional stage. MicroRNAs (miRNAs) are a fascinating new class of gene regulators that have been shown to have a critical role in neuroblastoma growth and progression. Chemotherapy, radiation and surgery are available treatment options for NB, but studies have reported that chemotherapy and radiation have severe side effects. NB, which is most commonly found in children, requires better treatment options suitable for their age group. Tolerating the side effects due to chemotherapy drugs and undergoing surgery is a little complicated in children. Hence, the use of miRNA-based drugs, such as anti-miRs, could be a better treatment option for treating NB patients of every age group.

In spite of the need, anti-miRs are studied only on a narrow scale which requires undivided attention. In vivo and in vitro studies could provide a base for molecular mechanisms and signalling pathways related to NB. Further, target genes are yet to be clearly analysed from various NB samples through random sampling to give a clear understanding of the therapeutic target. Large cohort studies are required to gather evidence on the above-said postulations. The use of nanoparticles for site-specific targeted drug delivery could reduce the chances of side effects and inflammation to bystander cells. Not only nanoparticles but also exosomes are studied as suitable vectors for targeted drug delivery. Exosomes are well known for their characteristics of protecting the DNAs, RNAs, proteins, miRNAs, etc. Hence, they can be used as vectors and used to target the particular site for treatment. NB requires studies on suitable biomarkers for early diagnosis and identification of potential targets for therapeutics. Therefore, there is an urgent requirement for many validated studies regarding new insights in NB.

## 7. Conclusions

Neuroblastoma (NB) is a rare type of solid tumour that arises in the adrenal glands. Despite recent therapeutic improvements, continuing clinical trials and basic scientific research, NB remains a difficult medical challenge with an uncertain clinical course and a poor overall prognosis for advanced-stage disease. With the discovery of microRNAs (miRNAs), a new layer of gene regulation has emerged, providing insights into the complicated pathophysiology of NB. Some of the down-regulated miRNAs, such as miR-3934-5p, miR-34a, miR-129, miR-362-5p, miR-205 and miR-338-3p, and up-regulated miRNAs, such as miR-380-5p, miR-15a, miR-15b, miR-16, miR-145, miR-200a, miR-1247, miR-149, miR-429, miR-203 and miR-9, have been studied for their role in NB disease progression and inhibition. These discoveries, on the other hand, give a very promising foundation for future research to investigate the role of miRNA in the therapy of neuroblastoma. In conclusion, the findings confirm the great clinical potential of miRNA inhibition in neuroblastoma and call for more intense research in this area.

## Figures and Tables

**Table 1:** The miRNAs and their function in neuroblastoma.

MiRNAs	Species	Target Gene	Function	References
MiR-380-5p	Mice		Decrease tumour size	Swarbrick et al. (2010) [37]
MiR-15a-5p, miR-15b-5p and miR-16-5p	Mice	*MYCN*	Tumor suppressive function	Chava et al. (2020) [38]
MiR-145	Human	*MTDH*	Reduce cell viability and increased apoptosis	Zhao et al. (2020) [39]
MiR-92	Human	*DKK3*	Stimulate or inhibit the canonical wnt pathway. Tumor suppressor	Haug et al. (2011) [40]
MiR-200a	Mouse	*AP-2γ*	Inhibits cell proliferation and tumour growth	Gao et al. (2014) [41]
MiR-429	Mouse		Role in cell proliferation, migration and invasion via NF-kB pathway	Zhou et al. (2020) [42]
MiR-1247	Human	*ZNF346*	Suppress cell proliferation, induce cell cycle G0/G1 phase arrest	Wu et al. (2018) [43]
MiR-149		*CDC42 and BCL2*	Inhibits cell proliferation and enhances chemosensitivity	Mao, et al. (2019) [44]
MiR-205	Human	*CREB1*	Inhibits neuroblastoma growth	Chen, S et al. (2018) [34]
MiR-3934-5p		*TP53INP1*	Induce apoptosis and inhibits cell viability	Ye, et al. (2019) [45]
MiR-34a	Human	*ATG5*	Inhibitory effect on proliferation, migration, invasion and autophagy	Cheng et al. (2019) [31]
MiR-129		*MYO10*	Inhibits tumour growth and potentiates chemosensitivity of neuroblastoma	Wang et al. (2018) [32]
miR-362-5p	Human	*(PI3K)-C2β*	Suppresses neuroblastoma cell growth and motility	Wu et al. (2015) [33]
miR-34a	Human	*HNF4α*	Prominent role in cell proliferation, migration and invasion	Li et al. (2019) [46]
miRNA-203	Human	*Sam68*	Role in malignant progression	Zhao et al. (2015) [39]
miR-338-3p	Human	*PREX2a*	Suppresses proliferation, invasion and migration	Chen et al. (2013) [38]
MiR-145	Human	*HIF-2α*	Represses migration, invasion and angiogenesis in neuroblastoma cell	Zhang et al. (2012) [47]
